# Overexpression of lncRNA HOXA11-AS promotes cell epithelial–mesenchymal transition by repressing miR-200b in non-small cell lung cancer

**DOI:** 10.1186/s12935-017-0433-7

**Published:** 2017-06-12

**Authors:** Jian-Hui Chen, Li-Yang Zhou, Suo Xu, Yu-Long Zheng, Yu-Feng Wan, Cheng-Ping Hu

**Affiliations:** 1 Department of Respiratory Medicine, Xiangya Hospital of Central South University (Key Site of National Clinical Research Center for Respiratory Disease), Changsha, 410008 Hunan China; 2 Department of Respiratory Medicine, Huai’an Second People’s Hospital, Huai’an, 223002 Jiangsu China; 3 Department of Emergency, The First People’s Hospital of Lianyungang, Lianyungang, 222002 Jiangsu China

**Keywords:** HOXA11-AS, Epithelial–mesenchymal transition, EZH2, DNMT1, miR-200b

## Abstract

**Background:**

Recent studies have verified that long noncoding RNAs (lncRNAs) involved in many biological functions and play crucial roles in human cancers progression, the study aimed to detect the association between long non-coding RNA HOXA11-AS and epithelial–mesenchymal transition (EMT) process in non-small cell lung cancer (NSCLC).

**Methods:**

The lncRNA HOXA11-AS expression levels were determined by quantitative real-time polymerase chain reaction (qRT-PCR) assays in 78 paired of tumor tissue and adjacent normal tissue samples in NSCLC patients. Kaplan–Meier survival curves and log-rank test was used to examine the association between lncRNA HOXA11-AS expression and the over survival time in NSCLC patients. Transwell invasion assay was performed to detect the cell invasion ability. QRT-PCR and western-blot analysis detected the mRNA and protein expression of EMT related transcription factors ZEB1/ZEB2, Snail1/2 and EMT marker E-cadherin and N-cadherin in NSCLC cells. RIP and Chromatin immunoprecipitation assays were performed to analyze the association between lncRNA HOXA11-AS and miR-200b expression in NSCLC cells.

**Results:**

The lncRNA HOXA11-AS expression levels were significantly higher in NSCLC tissues compared with adjacent normal tissues and higher HOXA11-AS expression levels had a poor prognosis in NSCLC patients. Furthermore, knockdown of lncRNA HOXA11-AS in A549 and H1299 cells dramatically inhibited cell invasive abilities. Besides, the transcription levels and protein levels of EMT related transcription factors ZEB1/ZEB2, Snail1/2, and EMT maker N-cadherin were down-regulated after lncRNA HOXA11-AS was knocked down, but the mRNA and protein expression levels of EMT maker E-cadherin was increasing in A549 and H1299 cells. The mechanistic findings showed demonstrated that HOXA11-AS interacted with EZH2 and DNMT1 and recruited them to the miR-200b promoter regions to repress miR-200b expression in NSCLC cells, which promoted cell EMT in NSCLC.

**Conclusions:**

Our results showed that up-regulation of lncRNA HOXA11-AS predicted a poor prognosis and lncRNA HOXA11-AS promoted cell epithelial–mesenchymal transition (EMT) by inhibiting miR-200b expression in NSCLC.

## Background

Lung cancer-associated mortality is the most common cause of cancer death worldwide [1]. Non-small cell lung cancer (NSCLC) accounts for approximately 85% in all lung cancer cases and more than half of all patients who had been diagnosed occurs tumor metastasis. More than 70% of NSCLC patients are diagnosed at advanced disease, the 5-year survival rate is just 15% [2, 3]. Hence, to investigate the molecular mechanism involving in NSCLC and obtain potential therapeutic target are urgently needed.

Long non-coding RNAs (lncRNAs) with no protein-coding are a recently characterized class of ncRNAs that are over 200 nucleotides in length [4, 5]. LncRNAs are involved in a variety of biological functions including molecular genetics, cellular processes, cell differentiation and cancer cell progression [6, 7]. Long noncoding RNAs (lncRNAs) are found to be related to different biological processes in non-small cell lung cancer (NSCLC). Such as, Long noncoding RNA AK126698 inhibits proliferation and migration of NSCLC cells by targeting Frizzled-8 and suppresses Wnt/β-catenin signaling pathway [8]. Downregulation of the long noncoding RNA GAS5-AS1 contributes to tumor metastasis in NSCLC [9]. Up-regulation of long non-coding HOTTIP functions as an oncogene by regulating HOXA13 in NSCLC [10]. Upregulation of long intergenic noncoding RNA 00673 promotes tumor proliferation via LSD1 interaction and repression of NCALD in non-small-cell lung cancer [11].

The long non-coding RNA HOXA11-AS (HOXA11 antisense RNA) is reported to participate in some cancer development including epithelial ovarian cancer [12], glioma [13], gastric cancer [14] and colorectal cancer [15]. LncRNA HOXA11-AS is also reported to be highly expressed in lung adenocarcinoma [3], however, the possible molecular mechanisms of lncRNA HOXA11-AS involved in NSCLC progression remained unknown.

In the study, we found that lncRNA HOXA11-AS expression was up-regulated in NSCLC tissues and patients who had increased lncRNA HOXA11-AS expression had a shorter survival time. Furthermore, we demonstrated that HOXA11-AS promoted cell invasive ability and epithelial–mesenchymal transition (EMT) process by repressing miR-200b via interacting with EZH2 and DNMT1 in NSCCL cells. Thus, our results showed that lncRNA HOXA11-AS may be a pivotal target for NSCLC therapy.

## Methods

### Patients and tissue samples

We collected paired NSCLC tissue and adjacent normal lung tissues from 78 cases of patients who underwent radical surgical resection between January 2010 and June 2014 at Huai’an Second People’s Hospital. No patient had received local or systemic treatment before any operation. All collected tissue samples were immediately frozen in liquid nitrogen and stored at −80 °C until RNA analysis. Written consent was obtained from each patient before tissue collection. The protocol was approved by the Institutional Research Ethics Committee of Huai’an Second People’s Hospital.

### Cell culture

The human NSCLC cells A549, H1299, 95D and normal human bronchial epithelial cells 16HBE cell lines were purchased from the Institute of Biochemistry and Cell Biology at the Chinese Academy of Sciences (Shanghai, China). Cells were cultured in the RPMI1640 medium (Hyclone, USA) and added with 10% fetal calf serum, 100 U/mL penicillin, and 100 μg/mL streptomycin, at 37 °C, high humidity, and 5% CO_2_.

### Cell transfection

2 × 10^5^ A549 and H1299 cells were seeded in 6-well plates and were incubated overnight, and then transfected using 100 nmol/L of small-interfering si-HOXA11-AS-1 or si-HOXA11-AS-2, miR-200b inhibitor and a negative control (NC) that were purchased from RiboBio (Guangzhou, China). Cells were transfected using Lipofectamine^®^ 3000 transfection reagent (Invitrogen, USA).

### Transwell assay

The invasive activity was detected by transwell invasion assays using 24-well transwell insert coated with Matrigel (8-μm pore size; Millipore). The 1 × 10^5^ cells that were transfected with si-HOXA11-AS or si-NC were seeded in serum-free medium and added on the upper chamber. In the lower chamber, cells were cultured with Medium supplemented with 10% fetal bovine serum. After 48 h of incubation, the cells on the upper surface of the filter were removed using a cotton swab, Cells in lower surface of the filter were fixed and stained with 0.5% crystal violet and counted under a light microscope.

### RNA isolation and quantitative real-time polymerase chain reaction (qRT-PCR)

The RNA was isolated from cell lines and lysed with Trizol (Life Technologies, Carlsbad, CA) and RNA isolation was performed according to the manufacturer’s protocol. RNA was reversed into cDNA using the PrimeScript™ RT reagent kit (Takara, Dalian, China) according to the manufacturer’s instructions. QRT-PCR assays were performed using SYBR^®^ Premix Ex Taq™ II (Takara) in the ABI PRISM^®^ 7500 real-time PCR system (Applied Biosystems, Foster City, CA, USA). GADPH were used as endogenous controls. The primer sequences for relative mRNA were HOXA11-AS-F:5′-GATTTCTCCAGCCTCCCTTC-3′ and HOXA11-AS-R:5′-AGAAATTGGACGAGACTGCG-3′. ZEB1-F:5′-TCCATGCTTAAGAGCGCTAGCT-3′, ZEB2-R:5′-ACCGTAGTTGAGTAGGTGTATGCCA-3′. ZEB2-F:5′-GGCGCAAACAAGCCAATCCCA-3′, ZEB2-R:5′-TTCACTGGACCATCTACAGAGGCTT-3′. Snail1-F:5′-CAAGGAATACCTCAGCCTGG-3′, Snail1-R:5′-ATTCACATCCAGCACATCCA-3′. Snail2-F:5′-CTACAGCGAACTGGACACACA-3′, Snail2-R:5′-GGAATGGAGCAGCGGTAGT-3′GAPDH-F:5′-TGGTATCGTGGAAGGACTCAT-3′, GAPDH-R:5′-GTGGGTGTCGCTGTTGAAGTC-3′. Data were collected and were analyzed using 2^−ΔΔCt^ method for quantification of the relative mRNA expression levels.

### Western blot analysis

Cells were transfected with si-HOXA11-AS-1 or si-HOXA11-AS-2 and si-negative control (NC) at 48 h and then cells were lysed using RIPA protein extraction reagent (Beyotime, Beijing, China) supplemented with phenylmethanesulfonyl fluoride (PMSF) (Beyotime, Beijing, China). About 40 μg of protein extracts were separated by 10% sodium dodecyl sulfate polyacrylamide gel electrophoresis (SDS-PAGE), transferred onto nitrocellulose membranes (Millipore, Bedford, MA, USA), and incubated with specific antibodies with ZEB1 (Santa Cruz Biotechnology Inc. Dallas, TX, USA), ZEB2 (Santa Cruz Biotechnology Inc. Dallas, TX, USA), Snail1 (Santa Cruz Biotechnology Inc. Dallas, TX, USA), Snail2 (Santa Cruz Biotechnology Inc. Dallas, TX, USA), E-cadherin (Cell Signaling, San Jose, CA, USA), N-cadherin (Cell Signaling, San Jose, CA, USA) and GAPDH (Cell Signaling, San Jose, CA, USA). The membrane was blocked with 5% skimmed milk in Tris-buffered saline and then incubated with second antibody for 1 h. An enhanced chemiluminescent (ECL) chromogenic substrate was performed to visualize the bands. The relative protein levels were normalized to GAPDH.

### RIP assay

RIP assays were carried out using the Magna RIP RNA-Binding Protein Immunoprecipitation Kit (Millipore, Billerica, MA, USA) following the manufacturer’s instructions in A549 and H1299 cells. Antibody for RIP assays of EZH2 (CST, USA) and DNMT1 (CST,USA) or control IgG (Millipore) were used in the study.

### Chromatin immunoprecipitation (ChIP) assays

CHIP assays was performed using a EZ-Magna ChIP kit (Millipore) according to manufacturer’s protocol, the A549 and H1299 cells were fixed with 4% paraformaldehyde and treated with glycine for 20 min to generate DNA–protein cross-links. Then, cells were lysed by lysis Buffer and Nuclear Lysis Buffer and sonicated to product chromatin fragments of 200–300 bp and cell lysates were immunoprecipitated with Magnetic Protein A Beads conjugated with EZH2 (CST, USA) and DNMT1(CST, USA) antibody.

### Statistical analysis

Statistical analysis was determined using the SPSS 18.0 software. All data were expressed as means  ±  standard deviation (SD). For comparisons between two samples, an unpaired two-tailed t test was performed. P values of less than 0.05 were considered as statistically significance.

## Results

### LncRNA HOXA11-AS expression levels are up-regulated in NSCLC tissues and cells

**Fig. 1:**
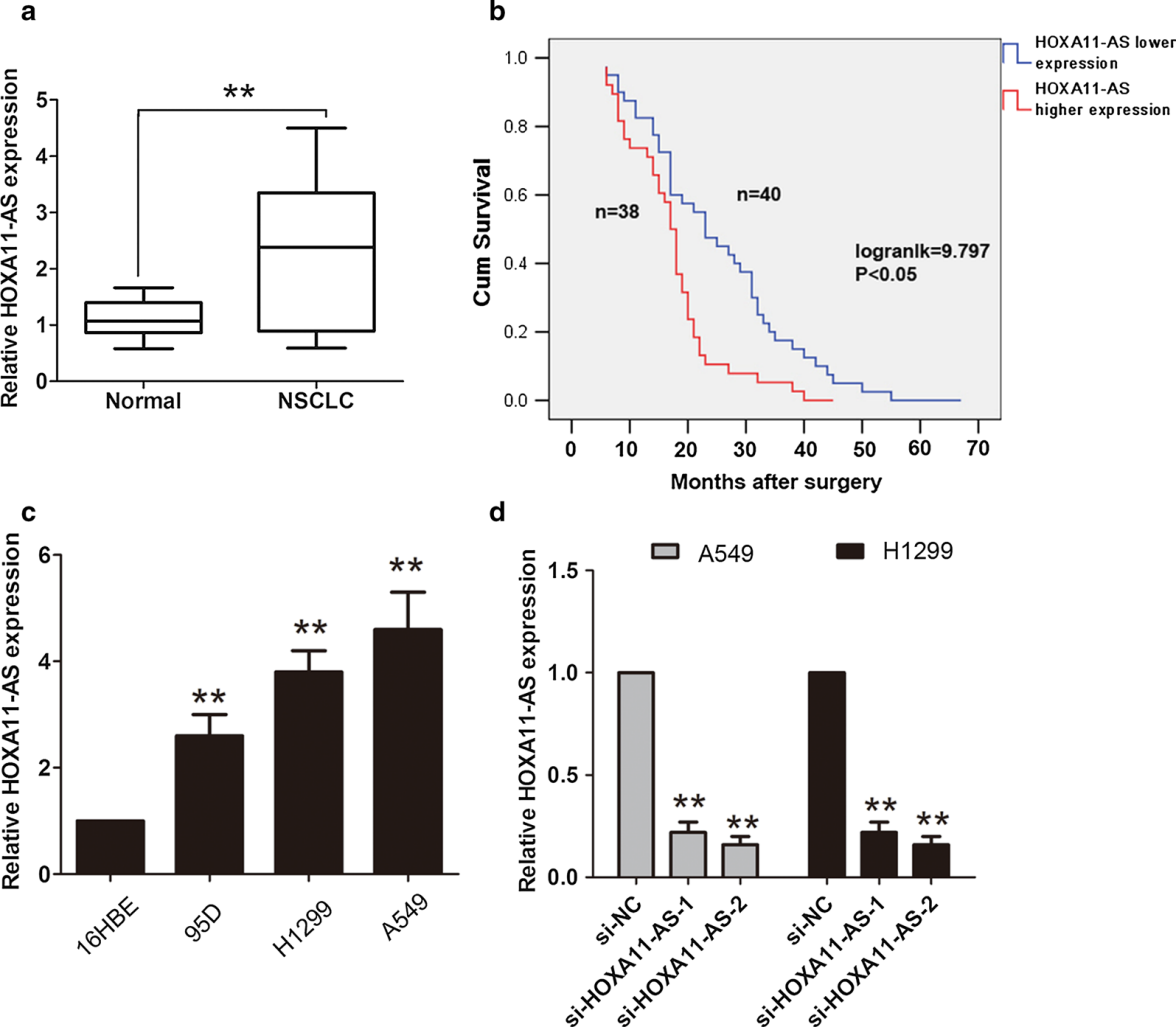
LncRNA HOXA11-AS expression levels were increased in NSCLC tissues. **a** The expression levels of lncRNA HOXA11-AS in human NSCLC tissues and adjacent normal tissues relative to GAPDH were determined by qRT-PCR assays (n = 78). **b** Kaplan–Meier survival curves and log-rank test was used to examine the association between lncRNA HOXA11-AS expression and the over survival time in NSCLC patients. **c** The expression levels of lncRNA HOXA11-AS in human NSCLC cells A549, H1299, 95D and normal human bronchial epithelial cells 16HBE to GAPDH were determined by qRT-PCR assays. **d** The expression levels of lncRNA HOXA11-AS in human NSCLC cells A549 and H1299 were determined by qRT-PCR assays after transfected with si-NC, si-HOXA11-AS-1 or si-HOXA11-AS-2. **P < 0.05, Mean ± SD was shown. Data show a representative of three independent experiments

To investigate the clinical significance of lncRNA HOXA11-AS expression in NSCLC, we collected 78 cases of primary NSCLC tissue samples and paired adjacent normal tissues. The lncRNA HOXA11-AS was analyzed by qRT-PCR assays and the results showed that lncRNA HOXA11-AS was significantly up-regulated in the NSCLC tissues, compared with the adjacent normal tissues (Fig. 1a). The median expression was 2.38-fold. According to the median expression of lncRNA HOXA11-AS, we classified the patients into two groups: higher lncRNA HOXA11-AS expression and lower lncRNA HOXA11-AS group. The association between lncRNA HOXA11-AS expression with clinicopathological factors showed that higher lncRNA HOXA11-AS expression levels were positively associated with the lymph node metastasis (P = 0.011) and TNM stage (P = 0.007) (Table 1). Moreover, we showed that the patients who had higher lncRNA HOXA11-AS had a worse survival compared with patients who had lower lncRNA HOXA11-AS (Fig. 1b, P < 0.05 log-rank test). We next examined lncRNA HOXA11-AS expression levels in three human NSCLC cells A549, H1299, 95D and a normal human bronchial epithelial cell 16HBE, the results discovered that lncRNA HOXA11-AS expression levels was higher compared with that in the 16HBE cell (Fig. 1c). Thus, these results demonstrated that lncRNA HOXA11-AS was up-regulated in NSCLC tissues and cells, and higher lncRNA HOXA11-AS predicted a poor prognosis in NSCLC patients.

**Table 1:** The association between lncRNA HOXA11-AS expression and clinicopathologic factors in NSCLC patients

Clinicopathologic factors	Patients number	LncRNA HOXA11-AS expression		*P*-value
		Lower (n = 40)	Higher (38)	
Sex				0.380
Male	47	26	21	
Female	31	14	17	
Age				0.621
≤60	45	22	23	
>60	33	18	15	
Smoking index				0.718
Yes	49	24	25	
No	29	16	13	
Tumor size (cm)				0.500
<3	40	22	18	
>3	38	18	20	
Histological differentiation				0.233
High and moderate	32	19	13	
Lower	46	21	25	
Histology				0.816
Squamous carcinoma	35	18	17	
Adenocarcinoma	33	16	17	
Other type	10	6	4	
Lymph node metastasis				0.011**
Negative	34	23	11	
Positive	44	17	27	
TNM stage				0.007**
I–II	43	28	15	
III	35	12	23	

*TNM* tumour-node-metastasis staging system

** P < 0.05

### Knockdown of lncRNA HOXA11-AS inhibits cell invasion ability in A549 and H1299 cells

**Fig. 2:**
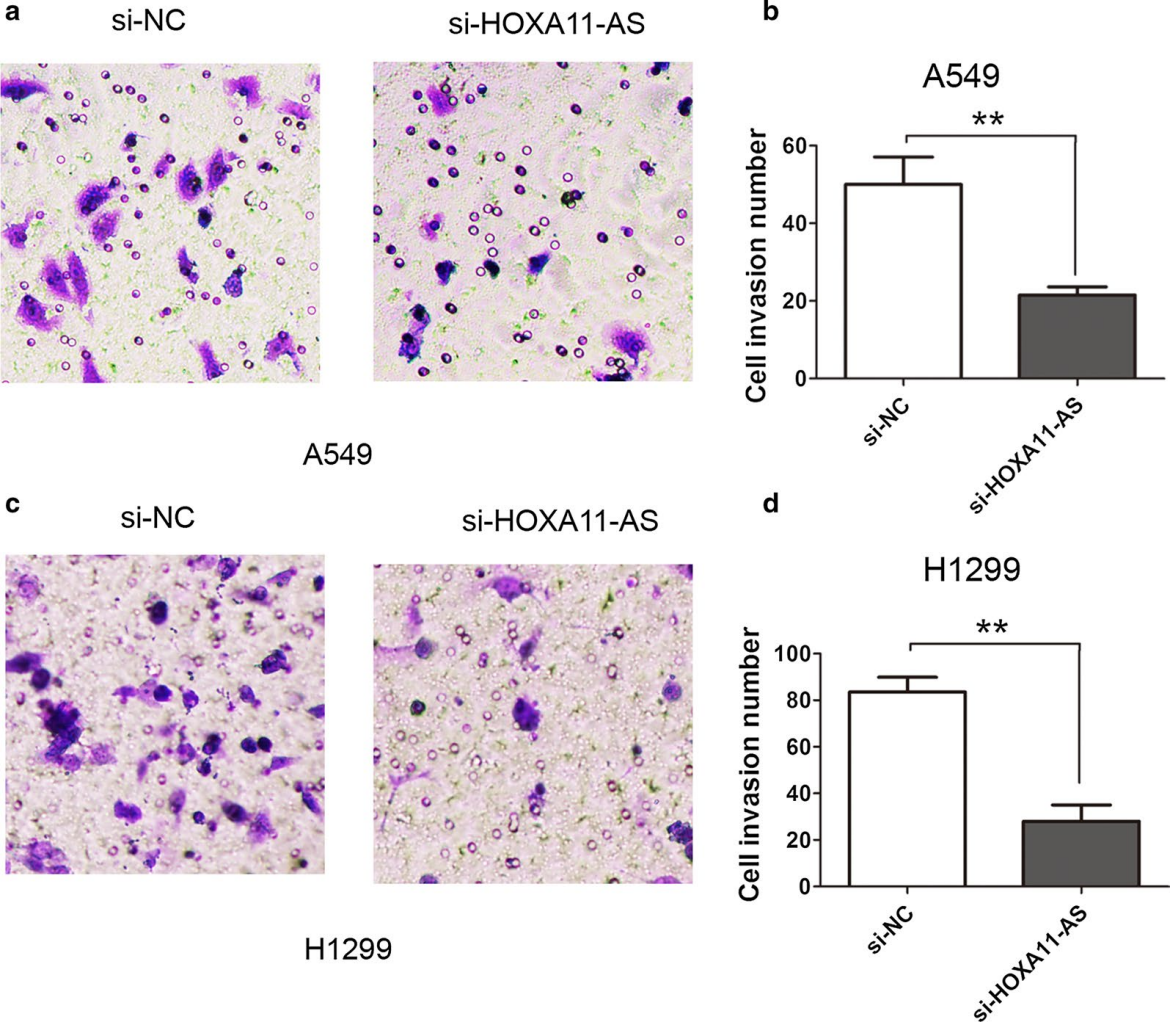
Knockdown of lncRNA HOXA11-AS inhibited cell invasion ability in A549 and H1299 cells. (**a**, **b**) Transwell assays were performed to determine the invasive abilities after lncRNA HOXA11-AS was knocked down in the A549 cells. (**c**, **d**) Transwell assays were performed to determine the invasive abilities after lncRNA HOXA11-AS was knocked down in the H1299 cells. **P < 0.05, Mean ± SD was shown. Data show a representative of three independent experiments

To assess the biological role of lncRNA HOXA11-AS in NSCLC cells, we down-regulated the expression of lncRNA HOXA11-AS in A549 cells and H1299 cells and determined the si-HOXA11-AS-2 had a better knockdown efficiency for lncRNA HOXA11-AS and were used in the following experiments (Fig. 1d). The transwell cell invasion was used to determine the cell invasion ability in A549 and H1299 cells. The results showed that silencing lncRNA HOXA11-AS significantly inhibited the cell invasion ability, compared with the si-NC group in A549 cells (Fig. 2a, b). Compared with the si-NC group in H1299 cells, the cell invasion ability also been inhibited when lncRNA HOXA11-AS was knocked down (Fig. 2c, d). Thus, our results showed that silencing HOXA11-AS suppressed the cell invasion ability in NSCLC.

### Knockdown of lncRNA HOXA11-AS suppresses cell EMT process in A549 and H1299 cells

**Fig. 3:**
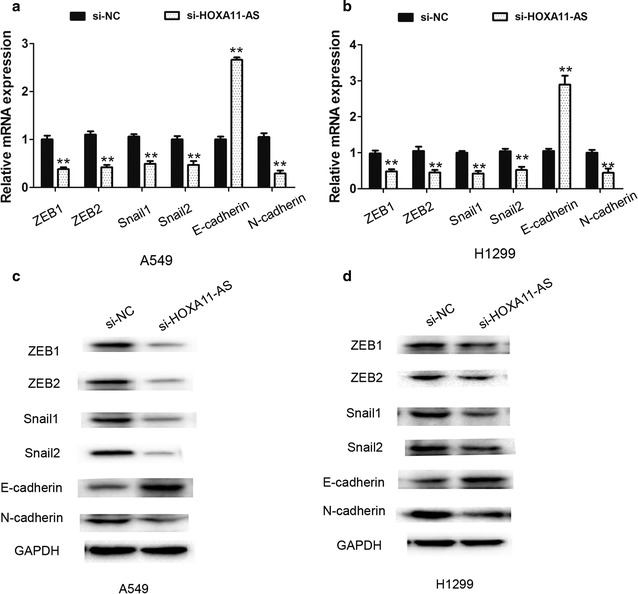
Knockdown of lncRNA HOXA11-AS inhibited cell EMT process in A549 and H1299 cells. (**a**, **b**) The mRNA expression levels of EMT related transcription factors ZEB1, ZEB2, Snail1, Snail2 and EMT marker E-cadherin, N-cadherin were determined by qRT-PCR assays in A549 cells and H1299 cells. (**c**, **d**) The protein expression levels of EMT related transcription factors ZEB1, ZEB2, Snail1, Snail2 and EMT marker E-cadherin, N-cadherin were determined by western-blot assays. **P < 0.05, Mean ± SD was shown. Data show a representative of three independent experiments

Epithelial–mesenchymal transition is an important factor in NSCLC cell invasion and metastasis [16]. Thus, we next determined whether EMT related transcription factors and markers were altered in our study. The mRNA and protein expression of ZEB1, ZEB2, Snail1, Snai2, E-cadherin and N-cadherin were analyzed by qRT-PCR and western blot assays. The results showed that the mRNA and protein expression of ZEB1, ZEB2, Snail1, Snail2 and N-cadherin were significantly decreased while E-cadherin expression was increased when lncRNA HOXA11-AS was knocked down in A549 cells, compared with the si-NC group (Fig. 3a, c). Compared with the si-NC group, the similar results also been found in H1299 cells after lncRNA HOXA11-AS silencing (Fig. 3b, d). Thus, these results demonstrated that lncRNA HOXA11-AS was involved in the EMT process and knockdown of lncRNA HOXA11-AS inhibits cell EMT process in NSCLC cells.

### LncRNA HOXA11-AS suppresses miR-200b expression by interacting with EZH2 and DNMT1 in NSCLC cells

**Fig. 4:**
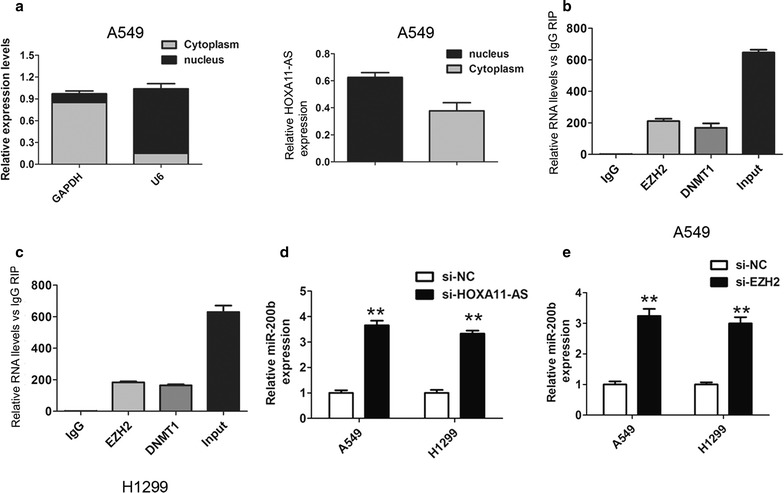
LncRNA HOXA11-AS bound to EZH2 and DNMT1 in NSCLC cells. (**a**) QRT-PCR was performed to analyze lncRNA HOXA11-AS expression in nuclear and cytoplasmic in A549 cells. U6 was used as a nucleus marker, and GAPDH was used as a cytosol marker. (**b**, **c**) RIP assays were performed and the co-precipitated RNA was subjected to qRT-PCR analysis of lncRNA HOXA11-AS in A549 and H1299 cells. The fold enrichment of lncRNA HOXA11-AS in EZH2 and DNMT1 RIP is relative to its matched IgG control. (**d**) The miR-200b expression levels were determined by qRT-PCR after silencing lncRNA HOXA11-AS in A549 and H1299 cells. (**e**) The miR-200b expression levels were determined by qRT-PCR after silencing EZH2 in A549 and H1299 cells. **P < 0.05, Mean ± SD was shown. Data show a representative of three independent experiments

To investigate the underlying mechanisms of lncRNA HOXA11-AS involved in EMT process in NSCLC. We detected the location of lncRNA HOXA11-AS in A549 and H1299 cells and found that lncRNA HOXA11-AS were enrich in nuclear and cytoplasmic fractions by qRT-PCR analysis (Fig. 4a). Many molecular mechanisms have been found to explain lncRNA-mediated epigenetically silencing related gene expression by interacting with EZH2 and DNMT1 [14]. Furthermore, we applied RIP assays to explore whether lncRNA HOXA11-AS interacted with EZH2 and DNMT1 in NSCLC cells. We found that lncRNA HOXA11-AS was enriched in anti-EZH2 and anti-DNMT1 antibodies RIP fraction group, compared with the IgG RIP fraction group (Fig. 4b, c). miR-200b had been found to suppressed cell EMT process and functioned as a target of EZH2, such as, DNMT1 and EZH2 mediated methylation silences the microRNA-200b/a/429 gene and promotes tumor progression [17]. Our results demonstrated that inhibition of lncRNA HOXA11-AS significantly up-regulated the miR-200b expression level in A549 and H1299 cells (Fig. 4d). Moreover, knockdown of EZH2 also increased the miR-200b expression in A549 and H1299 cells (Fig. 4e). Thus, the above results indicated that lncRNA HOXA11-AS could regulate the miR-200b expression interacted with EZH2 and DNMT1in NSCLC cells.

**Fig. 5:**
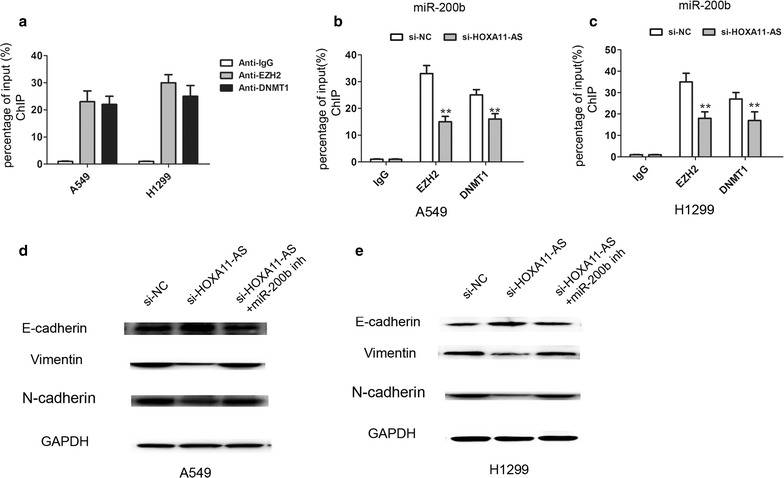
LncRNA HOXA11-AS epigenetically silenced miR-200b transcription by binding to EZH2 and DNMT1 in NSCLC cells. (**a**) ChIP assays of EZH2 and DNMT1 occupancy binding in the miR-200b promoter was detected by qRT-PCR assays in A549 and H1299 cells, IgG as a negative control. (**b**, **c**) ChIP assays of EZH2 and DNMT1 occupancy binding in the miR-200b promoter was detected by qRT-PCR assays after silencing lncRNA HOXA11-AS in A549 and H1299 cells, IgG as a negative control. (**d**, **e**) western blot analysis was used to determine the expression of E-cadherin, N-cadherin and Vimentin by transfecting si-NC, si-HOXA11-AS or si-HOXA11-AS+ miR-200b inhibitor into A549 and H1299 cells. **P < 0.05, Mean ± SD was shown. Data show a representative of three independent experiments

**Fig. 6:**
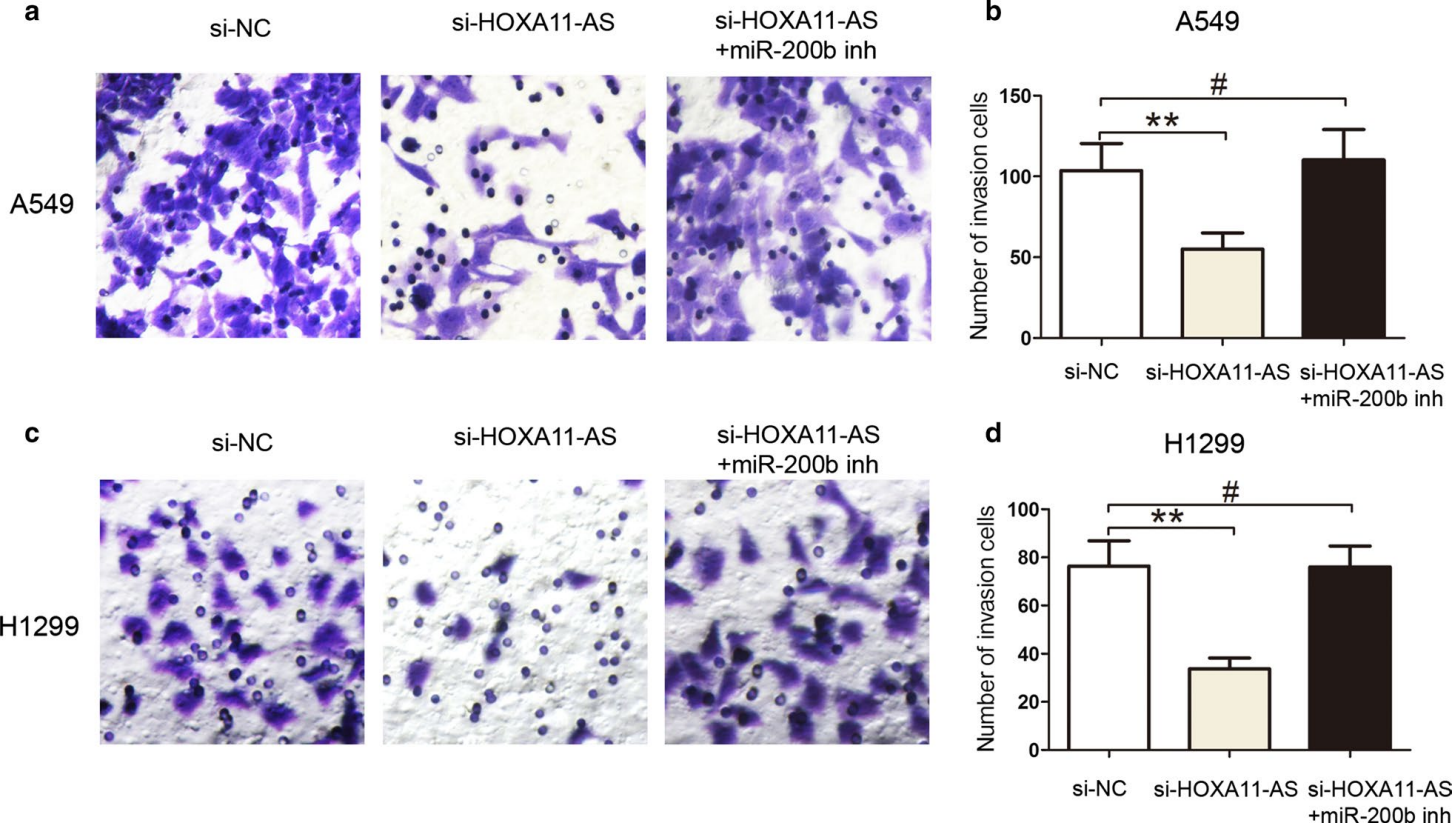
LncRNA HOXA11-AS promoted cell invasion abilities by inhibiting miR-200b in NSCLC. (**a**, **b**) Cells invasion capacities were determined by transwell invasion assays when si-NC, si-HOXA11-AS or si-HOXA11-AS+ miR-200b inhibitor were transfected into A549 cells. (**c**, **d**) Cells invasion capacities were determined by transwell invasion assays when si-NC, si-HOXA11-AS or si-HOXA11-AS+ miR-200b inhibitor were transfected into H1299 cells. **P < 0.05, Mean ± SD was shown. Data show a representative of three independent experiments. #, not statistical significance

Next, we explored whether lncRNA HOXA11-AS regulated miR-200b expression by recruiting EZH2 and DNMT1 to the promoter of miR-200b. The ChIP assays revealed that EZH2 and DNMT1 could bind to the promoter region of miR-200b in A549 and H1299 cells (Fig. 5a), and knockdown of significantly decreased the binding of EZH2 and DNMT1 to the promoter of miR-200b (Fig. 5b, c). The western blot analysis further showed that knockdown of lncRNA HOXA11-AS down-regulated EMT markers N-cadherin and Vimentin expression and up-regulated the E-cadherin expression, however, co-transfected with si-HOXA11-AS and miR-200b inhibitor dismissed the effects in A549 and H1299 cells (Fig. 5d, e). Moreover, knockdown of lncRNA HOXA11-AS inhibited cell invasion ability, however, co-transfected with si-HOXA11-AS and miR-200b inhibitor dismissed the effects in A549 and H1299 cells (Fig. 6a–d).Thus, these results confirmed that lncRNA HOXA11-AS promoted cell EMT and suppressed miR-200b expression by interacting with EZH2 and DNMT1 in NSCLC cells.

## Discussion

Some studies have showed that lncRNAs play important roles of regulating various cellular processes, such as proliferation, cell growth and apoptosis [18]. The lncRNA HOXA11-AS is reported to be higher expression highly expressed in lung adenocarcinoma [3]. In cervical cancer, the long non-coding RNA HOXA11 antisense is high expression and induces tumor progression and stemness maintenance [19]. Se et al. reports that lncRNA HOXA11 expression is lower in glioblastoma and is associated with treatment resistance and poor prognosis [13]. Sun et al. [14] reveals that lncRNA HOXA11-AS is up-regulated in gastric cancer and promotes cell proliferation and invasion of gastric cancer by scaffolding the chromatin modification factors PRC2, LSD1, and DNMT1. In the study, our results showed that lncRNA HOXA11-AS was higher expression in NSCLC and higher lncRNA HOXA11-AS expression levels were association with lymph node metastasis and TNM stage. In NSCLC patients, higher lncRNA HOXA11-AS expression predicted a poor prognosis.

Furthermore, we demonstrated that knockdown of lncRNA HOXA11-AS in NSCCL cells inhibited cell invasive ability and decreased the expression of EMT related transcription factors ZEB1, ZEB2, Snail1, Snail2 and EMT marker N-cadherin, but increasing the expression of E-cadherin. Some lncRNAs had been found to regulate the lung cancer invasion and metastasis by mediating the EMT process, such as, decreased BRAF activated non-coding RNA is associated with poor prognosis for NSCLC and promoted metastasis by affecting EMT [20]. Long non-coding RNA MALAT1 enhances brain metastasis by inducing EMT in lung cancer [21]. Down-regulation of long non-coding RNA FOXF1-AS1 regulates EMT, stemness and metastasis of NSCLC cells [22]. In the study, we revealed that lncRNA HOXA11-AS was involved in the NSCLC cell invasion and EMT process and knockdown of lncRNA HOXA11-AS inhibited the EMT by decreasing the expression of transcription factors ZEB1, ZEB2, Snail1, Snail2 and EMT marker N-cadherin and increased the expression of E-cadherin.

miR-200b has been found to be involved in the tumor invasion and EMT, such as, MicroRNA-200b suppresses cell invasion and metastasis by inhibiting the EMT in cervical carcinoma [23]. MicroRNA miR-200b affects cell proliferation, cell invasion and stemness of endometriotic cells by targeting ZEB1, ZEB2 and KLF4 [24]. miR-200b inhibits migration and invasion in NSCLC cells via targeting FSCN1 [25]. In the study, we demonstrated that lncRNAHOXA11-AS interacted with EZH2 and DNMT1 and inhibited miR-200b expression levels in NSCLC cells. LncRNAHOXA11-AS promoted cell EMT process by inhibiting miR-200b in NSCLC.

## Conclusion

In conclusion, we found that lncRNA HOXA11-AS was significantly up-regulated in NSCLC tissues, compared with adjacent normal tissues and higher lncRNA HOXA11-AS expression was association with poor prognosis in patients. Furthermore, we found that knockdown of lncRNA HOXA11-AS suppressed cell invasion and EMT phenomenon by repressing miR-200b in NSCLC. Thus, these results showed that lncRNA HOXA11-AS may be a pivotal target for NSCLC therapy.

## Abbreviations

NSCLC: non small cell lung cancer
qRT-PCR: quantificational real-time quantitative polymerase chain reaction
EMT: epithelial–mesenchymal transition
GAPDH: glyceraldehyde-3-phosphate dehydrogenase
